# Cigarette Smoke-Induced Lymphoid Neogenesis in COPD Involves IL-17/RANKL Pathway

**DOI:** 10.3389/fimmu.2020.588522

**Published:** 2021-02-05

**Authors:** Jing Xiong, Lu Zhou, Jieyu Tian, Xia Yang, Yunsong Li, Rong Jin, Yanqing Le, Yafei Rao, Yongchang Sun

**Affiliations:** ^1^ Department of Respiratory and Critical Care Medicine, Peking University Third Hospital, Beijing, China; ^2^ Department of Respiratory Medicine, Beijing Tongren Hospital, Capital Medical University, Beijing, China; ^3^ Department of Thoracic Surgery, Beijing Chest Hospital, Capital Medical University, Beijing, China; ^4^ Department of Immunology, School of Basic Medical Sciences, Peking University Health Science Center, Beijing, China

**Keywords:** chronic obstructive pulmonary disease, lymphoid follicles, interleukin 17, receptor activator of nuclear factor-κB ligand, RANK

## Abstract

IL-17 is critical in lung lymphoid neogenesis in COPD, but the cellular and molecular mechanisms remain to be elucidated. Receptor activator of nuclear factor-κB ligand (RANKL) functions in lymphoid follicle formation in other organs, whether it is involved in IL-17A–dependent lymphoid neogenesis in COPD is unknown. To elucidate the expression and functional role of IL-17A/RANKL pathway in COPD. We first quantified and localized RANKL, its receptor RANK and IL-17A in lungs of patients with COPD, smokers and non-smokers. Next, IL-17A^−/−^ and wild-type (WT) mice were exposed to air or cigarette smoke (CS) for 24 weeks, and lung lymphoid follicles and RANKL-RANK expression were measured. Lastly, we studied the *in vitro* biological function of RANKL pertaining to lymphoid neogenesis. We found that the expressions of RANKL-RANK and IL-17A, together with lymphoid follicles, were increased in lung tissues from patients with COPD. In WT mice exposed to CS, RANKL-RANK expressions were prominent in lung lymphoid follicles, which were absent in IL-17A^−/−^ mice exposed to CS. In the lymphoid follicles, RANKL^+^ cells were identified mostly as B cells and RANK was localized in dendritic cells (DCs). *In vitro* IL-17A increased the expressions of RANKL in B cells and RANK in DCs, which in turn responded to RANKL stimulation by upregulation of CXCL13. Altogether, these results suggest that B lymphocyte RANKL pathway is involved in IL-17A–dependent lymphoid neogenesis in COPD.

## Introduction

Chronic obstructive pulmonary disease (COPD) is a heterogeneous disease that relates to cigarette smoking (1). The pathological features of COPD are small airway inflammation and pulmonary parenchymal destruction, which lead to emphysema (2). Recent studies have shown that adaptive immunity may play a role in the pathogenesis of COPD (3), as evidenced by the formation of tertiary lymphoid follicles in patients and cigarette smoke (CS)-exposed models (4–6), which explains at least in part why the disease is progressing even after cessation of smoking. However, the molecular mechanisms underlying lung lymphoid neogenesis associated with cigarette smoking have not been fully elucidated.

Interleukin 17 (IL-17) is a pro-inflammatory cytokine that is mainly secreted by T-helper 17 cells. In addition, it plays a vital role in a variety of autoimmune diseases (7). Th17 cells and IL-17 increased in COPD lung tissues and were associated with pathological changes and lung function (8, 9). Studies have shown that IL-17 is critical in the formation of tertiary lymphoid tissues (10), including that induced by CS exposure (11), probably by upregulating the expression of B cell chemokines CXCL12/13 (10, 11).

Receptor activator of nuclear factor-κB (RANK) and its ligand (RANKL) have originally been described for their key roles in bone metabolism, and later in immune regulation (12–15). RANKL gene knockout mice showed a significant decrease in lymphoid follicle formation in small intestines, and a failure of stromal cells to produce CXCL13, a potent chemotactic factor for B lymphocytes (16). RANKL functions in lymph node homeostasis by stimulating an equilibrated proliferation of all stromal cell subsets concomitant with increased lymphocyte recruitment (17). Previous studies with osteoclasts have revealed that IL-17A can induce the expression of RANKL in fibroblasts or osteoblasts and indirectly drive bone destruction (18). Whether IL-17 induces RANKL expression and therefore contributes to lung lymphoid neogenesis in COPD has not been described.

In this study, we hypothesized that IL-17A/RANKL pathway was involved in lung lymphoid follicle formation in COPD associated with CS exposure. We showed that IL-17A, RANKL, and its receptor RANK were highly expressed in the lung tissues and lymphoid follicles from smokers with COPD. We further demonstrated that lymphoid follicles and RANKL-RANK expression were absent in IL-17A knockout mice exposed to long-term CS. RANKL expression, which was localized mostly in B cells in COPD patients and the CS-exposed model, was induced by IL-17A *in vitro*. While dendritic cells (DCs), the RANK-expressing cell in lymphoid follicles, responded to RANKL stimulation by expressing CXCL13. These results reveal a potentially important role of IL-17A/RANKL pathway and a self-perpetuating mechanism involving B cell-DC interaction in COPD lymphoid neogenesis.

## Methods

### Human Subjects

Lung resection samples were obtained from smokers with COPD (n=28), smokers (n=26) and never-smokers (n=22) with normal lung function, all undergoing surgery for solitary lung tumors at Peking University Third Hospital and Beijing Chest Hospital. Lung tissue at maximum distance from the pulmonary lesions and without signs of retro-obstructive pneumonia or tumor invasion was collected by a pathologist. Characteristics of the study subjects are shown in **Table 1**. The study was approved by the Ethics Committee of Peking University Third Hospital and Beijing Tongren Hospital, Capital Medical University. Signed informed consent was obtained from all the study subjects.

**Table 1:** Characteristics of the study population (n = 76).

	Never-smokers	Smoker	COPD					*P* Value (ANOVA)
			GOLD I		GOLD II	GOLD III	GOLD IV	
Subjects (n)	22	26	7	17		4	0	
Male/Female (n)	6/16	23/3	18/10					<0.001*
Age (years)	54.74 ± 2.24	61.38 ± 1.87	58.44 ± 1.99					NS
Body mass index	24.89 ± 0.78	24.75 ± 0.76	23.49 ± 0.68					NS
Smoker/ex-smoker	N/A	19/7	22/6					
Pack-years	N/A	51.63 ± 6.07	58.00 ± 10.14					NS^#^
FEV1, % predicted	92.23 ± 3.82	93.05 ± 2.47	69.32 ± 3.88					<0.001
FEV1/FVC	83.13 ± 1.61	77.69 ± 1.01	62.05 ± 1.31					<0.001

ANOVA, analysis of variance; COPD, chronic obstructive pulmonary disease; GOLD, Global Initiative for Chronic Obstructive Lung Disease; N/A, not applicable; NS, not significant.

Values are mean ± SEM.

*Categorical variables were analyzed with Chi-Square tests.

^#^is used Student’s t test.

### Animals and Experimental Design

Six to 8 week-old female C57BL/6 mice were supplied by Beijing Vital River Laboratory and female IL-17A^−/−^ mice (6–8 weeks old) on C57BL/6 background (generated by Dr. Yoichiro Iwakura, and provided by Dr. Huanzhong Shi, Capital Medical University) were bred in-house. Food and water were provided *ad libitum*. All mice were housed with a light-dark cycle of 12 h, under specific pathogen-free conditions. All *in vivo* manipulations were approved by the Ethics Committee of Peking University Third Hospital and Beijing Tongren Hospital, Capital Medical University.

12 wild-type (WT) mice and 12 IL-17A^−/−^ mice were exposed to CS using a nose-only, directed flow inhalation and smoke-exposure system (SG-300, SIBATA, Tokyo, Japan). CS exposure parameters: cigarettes (Baisha cigarettes with filter; Hunan China. Tar 11 mg, nicotine 0.9 mg, CO 12 mg), suction 20 ml smoke per 8 s, two times a day for 50 min with 20 min smoke-free intervals, 5 days a week for 24 weeks. An optimal smoke/air ratio of 1:9 was obtained. Control mice were exposed to room air only. Mice were anesthetized by 1% pentobarbital sodium (70 mg/kg, intraperitoneal; Sigma-Aldrich) and sacrificed by exsanguination. The left lung tissues were then collected for histopathology analysis and right lung tissues were stored in −80°C for western blot analysis.

### Immunohistochemistry Staining

The lung tissues obtained from the patients or mice were fixed in 4% paraformaldehyde and embedded in paraffin. After dewaxing and hydration, lung sections (5 μm thick) were incubated in 0.3% hydrogen peroxide for 15 min and then incubated in citrate buffer 5 mM at pH 6.0 in a microwave oven for antigen retrieval. Afterward, sections were blocked with goat serum (ZLI-6056, ZSGB-Bio, Beijing, China) and incubated overnight with the primary antibody (antibodies presented in **Table 2**). Sections were subsequently incubated with horseradish peroxidase (HRP) conjugated goat anti mouse IgG (PV-6002, ZSGB-Bio) for 30 min. Immunoreactivity was visualized with DAB Detection System kit (ZLI-9018, ZSGB-Bio). Images were captured using Olympus BX51 microscope and analyzed by image-pro plus 6.0 software.

**Table 2:** Antibodies.

Antigen	Supplier	Dilution
IL-17A	Abcam (Cambridge, United Kingdom)	1:200
RANKL	Abcam (Cambridge, United Kingdom)	1:150
RANK	Abcam (Cambridge, United Kingdom)	1:150
CD20	Abcam (Cambridge, United Kingdom)	1:300
CD11c	Abcam (Cambridge, United Kingdom)	1:100
B220	Abcam (Cambridge, United Kingdom)	1:200
CD21	Abcam (Cambridge, United Kingdom)	1:250
CD3	Abcam (Cambridge, United Kingdom)	1:100
CXCL13	Abcam (Cambridge, United Kingdom)	1:250

Cell counts were calculated and standardized to the number of positive cells/mm^2^ of the area of interest (cluster, follicle or subepithelium). Twenty fields were randomly selected under 400× microscopy, and the number of positive cells was calculated per mm^2^.

### Quantification of Lymphoid Aggregates

Dense accumulations of 50 or more lymphomononuclear cells were defined as lymphoid follicles, whereas accumulations of fewer than 50 cells were defined as lymphoid aggregates. The number of lymphoid aggregates in lung tissue sections of mice was assessed as described by others (6, 19). Briefly, paraffin embedded sections of the left lung were stained with H&E. Lymphoid follicles (aggregates) in the tissue area surrounding the airways were expressed as counts relative to the numbers of airways per lung section, while those in the pulmonary parenchyma were expressed as counts relative to the area per lung section.

### Immunofluorescence Staining

Confocal microscopy was applied to evaluate the coexpression of RANKL and the B cell marker B220 (mouse) or CD20 (human), the coexpression of RANKL and the T cell marker CD3, and the coexpression of RANK and the dendritic cell marker CD21 (mouse) or CD11c (human). Briefly, after dewaxing and hydration, sections were submerged in citrate buffer (pH 6.0) in a microwave oven for antigen retrieval, blocked with goat serum and then incubated overnight with the primary antibody (antibodies presented in **Table 2**). Sections were subsequently incubated with the secondary antibody (goat anti-rabbit IgG conjugated with AlexaFluor 488 1:500 and goat anti-mouse IgG conjugated with AlexaFluor 594 1:500, Jackson ImmunoResearch, West Grove, PA) for 30 min 37°C. Slides were stored at 4°C and analyzed within 24 h. Immunofluorescence was evaluated with a confocal microscopy (TCS-SP8, Leica, Wetzlar, Germany).

### Western Blot Analysis

RANKL and RANK protein levels were determined in lung tissue of mice using Western Blotting. Proteins (30 μg) were resolved in 10% SDS-polyacrylamide electrophoresis gels and transferred to PVDF membrane (Merck-Millipore, Solna, Sweden), which were then blocked and incubated with antibodies: RANK (Abcam), RANKL (Abcam), and β-actin (Abcam). After incubation with HRP-conjugated goat anti-mouse IgG antibody, the immunoreactive bands were detected using the enhanced chemiluminescence from Millipore Company (Bedford, MA). Quantitative image analysis was performed with Image J software (NIH, Bethesda, MD). Results are expressed as relative densities.

### Purification and Culture of B Cells and Bone Marrow-Derived Dendritic Cells

Mice were killed with spinal dislocation and single-cell suspensions of splenocytes were obtained by dispersing spleen tissues through a 300-mesh stainless steel screen. Erythrocytes were removed by red cell lysis buffer (Miltenyi Biotech, Bergisch Gladbach, Germany). B cells were further isolated from splenocytes by negative selection of CD19+ cells with magnetic microbeads (Miltenyi Biotech). Purified B cells were stimulated with recombinant IL-17A (R&D systems, Minneapolis, MN, 100 ng/ml) and anti-CD40 (R&D systems, 2.5 μg/ml), with Phorbol-12-Maristate-13-Acetate (PMA) (Abcam, 50 ng/ml) at 37°C, 5% CO_2_ for 48 h, and anti-IL-17RC (R&D systems, 2.5 μg/ml) and anti-IL-17RA (R&D systems, 1.5 μg/ml) were used to neutralize IL-17A receptors, RANKL expression was detected by flow cytometry.

The preparation of bone marrow dendritic cells (BMDCs) was performed as previously described with slight modification (20). Bone marrow cells from the femurs and tibias of female C57BL/6 mice (6–8 weeks old) were depleted of red cells with lysis buffer and cultured in RPMI 1640 (Gibco, Grand Island, NY) medium supplemented with 10% FBS, recombinant murine granulocyte macrophage colony stimulating factor (GM-CSF) (R&D systems, 10 ng/ml), interleukin (IL)-4 (R&D systems, 1 ng/ml), 100 units/ml penicillin and 100 μg/ml streptomycin. After incubation for 24 h, the medium containing non-adherent cells was removed and replaced with fresh medium as described above. After 7 days of culture, the cells were harvested.

BMDCs were stimulated with recombinant IL-17A (100 ng/ml), recombinant RANKL (R&D systems, 100 ng/ml) or LPS (for RANK expression; Sigma-Aldrich, St. Louis, MO, 1 µg/ml) at 37°C, 5% CO2 for 48 h. RANK expression was detected by flow cytometry, and CXCL13 mRNA was measured using real-time PCR.

### Flow Cytometry

At the termination of cell culture, cells were harvested and followed by incubation with fluorescence conjugated antibodies: APC-conjugated anti-mouse CD19 antibody (eBioscience, San Diego, CA), PE-conjugated anti-mouse RANKL antibody (eBioscience); APC-conjugated anti-mouse CD11c antibody (eBioscience), PE-conjugated anti-mouse RANK antibody (eBioscience). At least 10,000 cells were counted for each sample using Beckman Coulter Gallios (Brea, CA). Cells suspended in medium without any stimulus was used as blank control for positive cell gating.

### Reverse Transcription-Polymerase Chain Reaction (RT-PCR)

Total RNA was extracted from the total lung tissues of mice and cultured cells using TRIZOL (Life Technology, Rockville, MD) following manufacturer’s instructions. Isolated mRNA (1 µg each) was reverse transcribed into cDNA using the Reverse transcription system in the presence of oligo dT primers (Promega, Madison, WI). Real-time PCR was carried out in a 20 µl reaction system using SYBR Green One-Step qRT-PCR Kit (Tiangen, Beijing, China) in Applied Biosystems (Foster City, CA).

The primer sequences used for the amplification were as follows: GAPDH, 5′-AAATGGTGAAGGTCGGTGTGAAC-3′ (sense) and 5′-CAACAATCTCCACTTTGCCACTG-3′ (antisense); CXCL13, 5′-ACTCCACCTCCAGGCAGAATG-3′ (sense) and 5′-AAGTTTGTGTAATGGG CTTCCAGA-3′ (antisense). The real-time PCR conditions were: 95°C for 15 min, followed by 40 cycles of 95°C for 10 s and 60°C for 32 s. Results were presented as fold changes relative to GAPDH reference.

### Statistical Analysis

Statistical analysis was performed with SPSS20.0 (IBM, Chicago, IL). Group data are expressed as mean ± SEM. Statistical significance of differences in group mean values was detected by one-way ANOVA followed by Bonferroni *post hoc* tests (equal variances assumed) or Dunnett’s T3 *post hoc* tests (equal variances not assumed). Categorical variables were analyzed with Chi-Square tests. Two groups comparison was performed using Student’s *t* test. P values less than 0.05 were considered to be significant.

## Results

### RANKL-RANK and IL-17A Were Expressed Within Lymphoid Follicles in COPD Patients

Tertiary lymphoid follicles were found in the lungs from patients with COPD, more prominent in patients with advanced disease (4, 5). In our study, because few patients with very severe COPD received lung surgery, we collected lung samples only from patients with grade I-III COPD.

**Figure 1:**
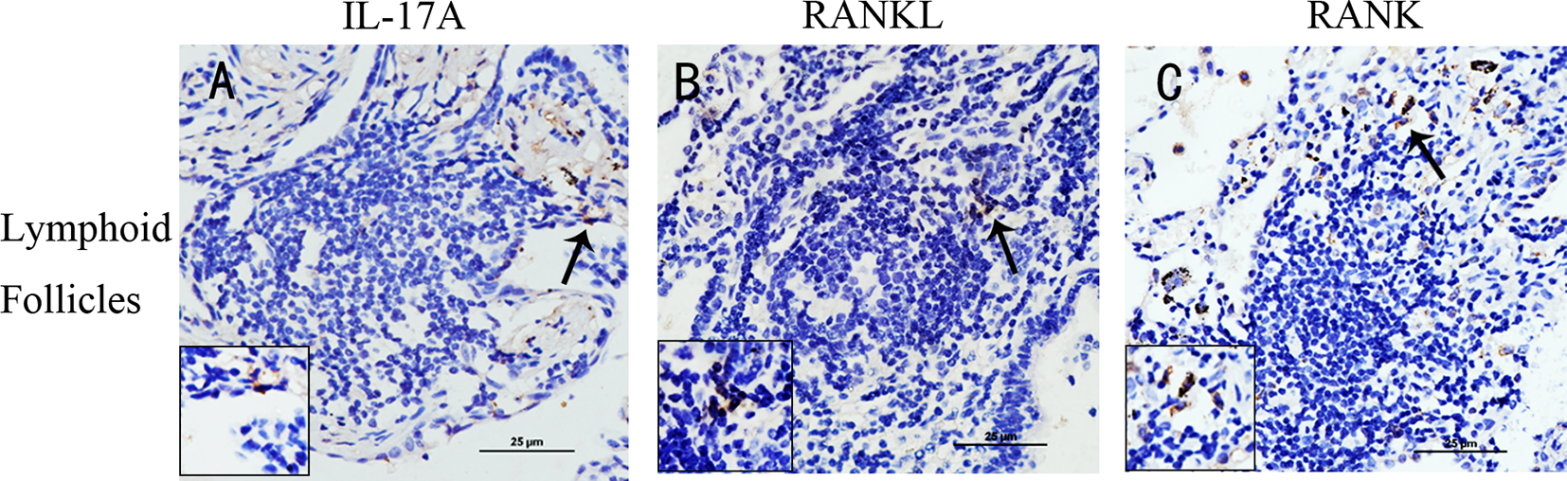
IL-17A, RANKL, and RANK are expressed in lung lymphoid follicles of COPD. Immunohistochemical detection of **(A)** IL-17A, **(B)** RANKL, and **(C)** RANK (3,39-diaminobenzidine (DAB), brown) in lymphoid follicles in lung tissues from patients with COPD. Sections were counterstained with Mayer hematoxylin (blue). Arrows indicate positive cells. Scale bar = 25 μm.

To examine whether RANKL/RANK, together with IL-17, were expressed in COPD, we performed immunostaining of lung tissues from COPD patients, smokers and non-smokers with normal lung function. IL-17A^+^ cells were identified within lymphoid follicles/aggregates in lung tissue sections from patients with COPD (**Figures 1A**), in agreement with previous findings (11). More notably, RANKL^+^ cells and RANK^+^ cells were also identified within these lymphoid follicles/aggregates (**Figures 1B**, **1C**), suggesting that RANKL might be involved in the formation of lymphoid follicles.

### Expressions of RANKL-RANK and IL-17A Were Increased in Lung Tissues From COPD Patients

**Figure 2:**
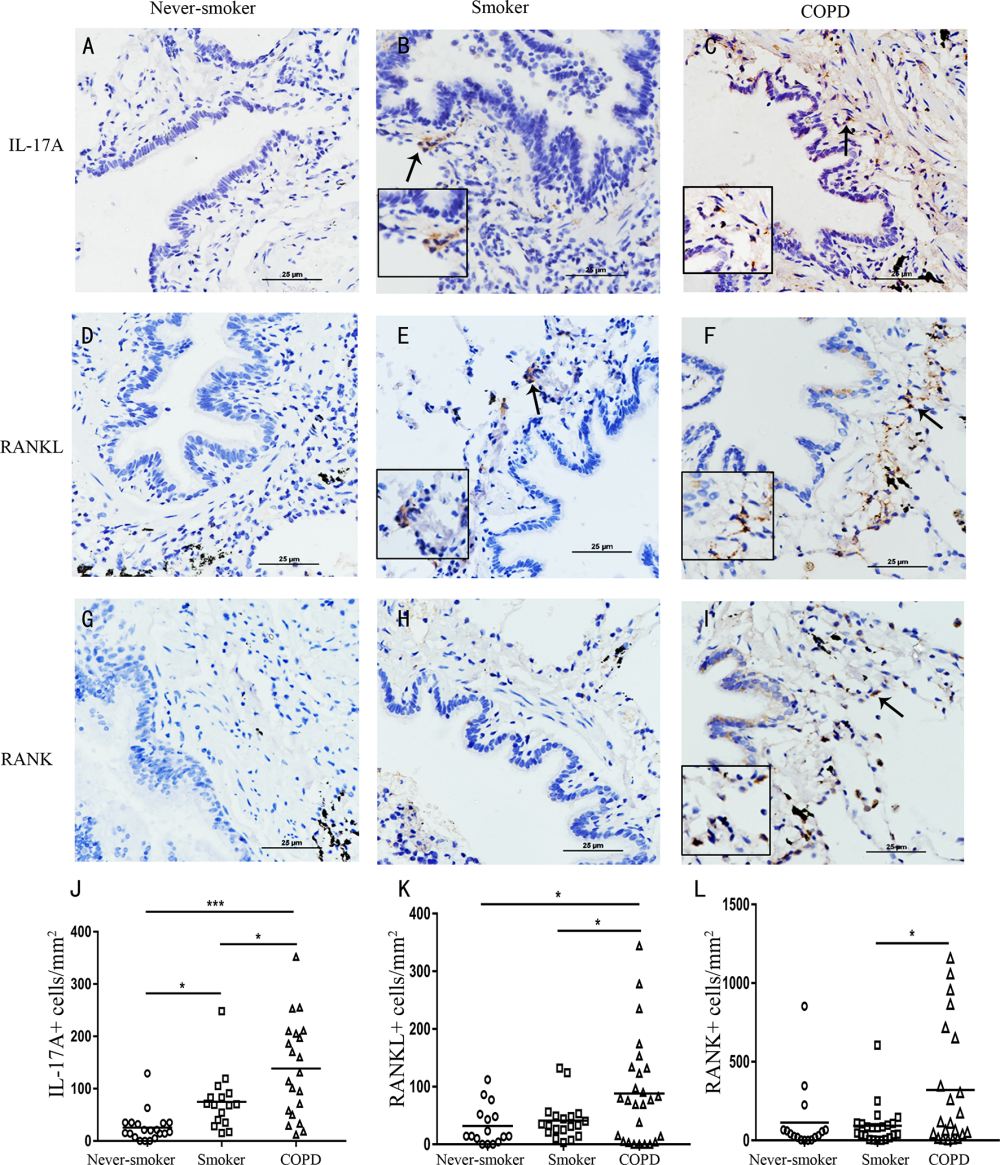
Elevated expression of IL-17A, RANKL, and RANK in COPD. IL-17A in **(A)** never-smoker, **(B)** non-COPD smokers, and **(C)** smokers with COPD. RANKL in **(D)** never-smoker, **(E)** non-COPD smokers, and **(F)** smokers with COPD. RANK in **(G)** never-smoker, **(H)** non-COPD smokers, and **(I)** smokers with COPD. Positive immunoreactivity was visualized by immunohistochemistry and DAB detection chromogen (brown). Sections were counterstained with Mayer’s hematoxylin (blue). Arrows indicate positive cells. Scale bars = 25 μm. Number of IL-17A **(J)**, RANKL **(K)** and RANK **(L)** positive cells per square millimeter. Horizontal lines indicate mean value. n = 16-25; *P < 0.05; ***P < 0.001.

We found outside of lymphoid follicles, compared with never-smoker group, IL-17A was also more evidently detected in the cytoplasm of cells within the small airways, bronchiolar walls, and the lung parenchyma in smoker group and COPD group (**Figures 2A–C**). RANKL was also more obviously detected in the cytoplasm of cells within the small airways, bronchiolar walls, and the lung parenchyma in COPD group compared with never-smoker group or smoker group (**Figures 2D–F**), and a similar pattern was observed for RANK expression (**Figures 2G–I**).

Next, image analysis was performed to quantify the IL-17A^+^ cells, RANKL^+^ cells and RANK^+^ cells. The numbers of IL-17A^+^ cells and RANKL^+^ cells were increased, and significantly higher in COPD compared with never-smoker subjects or smoker subjects without airway obstruction (*P*<0.05) (**Figures 2J, K**). The numbers of RANK^+^ cells were increased and significantly higher in COPD compared with smokers without COPD (*P*<0.05) (**Figures 2L**). Thus, these data demonstrate that COPD is associated with an increased expression of RANKL-RANK, concomitant with IL-17A in the lung.

### Cigarette Smoke-Induced Lymphoid Neogenesis and RANKL/RANK Expression in the Lung Were Dependent on IL-17A

**Figure 3:**
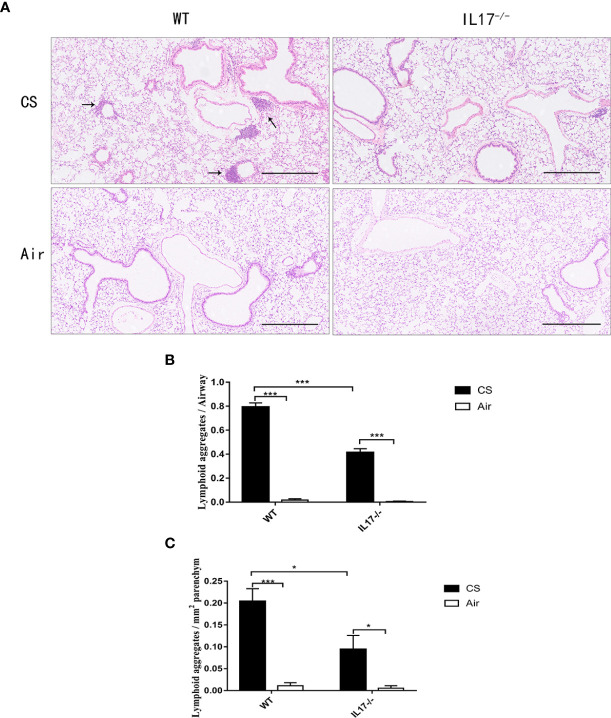
CS-induced lymphoid neogenesis is dependent on IL-17. **(A)** Wild type (WT) and IL17^−/−^ mice were exposed to cigarette smoke (CS) two times daily, 5 days a week for 24 weeks or air. Arrows indicate lymphoid follicles. Scale bar = 450 µm. **(B)** Number of CS-induced lymphoid aggregates per airway and **(C)** number of CS-induced lymphoid aggregates per mm^2^. Bars indicate mean value, and error bars indicate SEM. n = 6; *P <0.05; ***P <0.001.

Because IL-17 is critical in the formation of tertiary lymphoid tissue in the lung (10, 11), and IL-17A was elevated in COPD, we firstly investigated whether CS-induced lymphoid neogenesis required IL-17A using C57BL/6 WT mice and IL-17A^−/−^ mice. After exposure to CS for 24 weeks, highly organized lymphoid follicles were evident in WT mice, but not in IL-17A^−/−^ mice (**Figure 3A**). The ratio of the number of lymphoid aggregates to the number of airways was significantly lower in CS-exposed IL-17A^−/−^ mice compared with CS-exposed WT animals (**Figure 3B**). Not surprisingly, the number of lymphoid aggregates per square millimeter in every section was significantly lower in IL-17A^−/−^ mice exposed to CS compared with CS-exposed WT animals (**Figure 3C**). These findings confirmed that IL-17A was critical in the formation of CS-induced lymphoid follicles (11).

**Figure 4:**
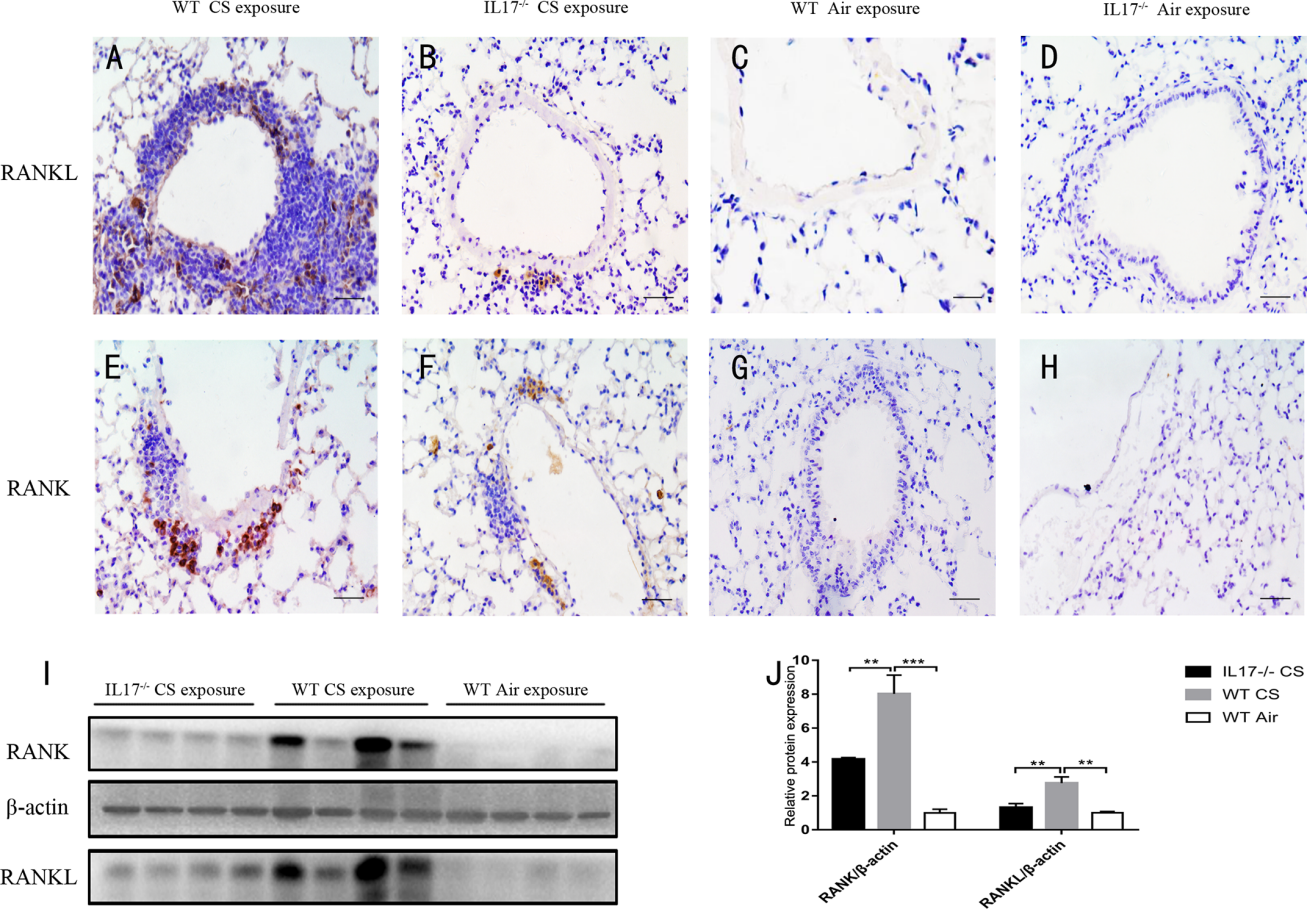
CS-induced expression of RANKL and RANK is dependent on IL-17A. Immunohistochemical detection of RANKL (DAB, brown) in **(A)** CS-exposed WT mice, **(B)** CS-exposed IL17^−/−^ mice, **(C)** air-exposed WT mice and **(D)** air-exposed IL17^−/−^ mice. Immunohistochemical detection of RANK (DAB, brown) in **(E)** CS-exposed WT mice, **(F)** CS-exposed IL17^−/−^ mice, **(G)** air-exposed WT mice and **(H)** air-exposed IL17^−/−^ mice. Sections were counterstained with Mayer hematoxylin (blue). Scale bar = 50 μm. **(I)** RANKL and RANK are detected with Western Blot in whole lung tissues from WT and IL17^−/−^ mice exposed to air or CS. **(J)** Expression of RANKL and RANK relative β-actin in whole lung tissues of mice. Bars indicate mean value, error bars indicate SEM. n = 4; **P <0.01; ***P<0.001.

Next we investigated whether IL-17A was critical for the expression of RANKL-RANK in the context of lymphoid follicle formation induced by CS. After exposure to CS for 24 weeks, WT mice showed markedly increased expression of RANKL (**Figures 4A, C**), which was most prominent in lymphoid follicles, although scarce expression was observed elsewhere. Notably, the expression of RANKL was almost absent in CS-exposed IL-17A^−/−^ mice (**Figures 4B, D**), as was in WT mice exposed to air. The protein level of RANKL, detected by Western blotting, was also reduced significantly in lung tissues from CS-exposed IL-17A^−/−^ mice compared with CS-exposed WT mice (**Figures 4I, J**). Interestingly, a similar expression pattern was observed for RANK (**Figures 4E-H, I**, **J**). Thus, IL-17A is critical in RANKL-RANK expression in lung lymphoid follicles induced by CS exposure.

### Cellular Localization of RANKL and RANK in COPD and CS-Exposed Mice

**Figure 5:**
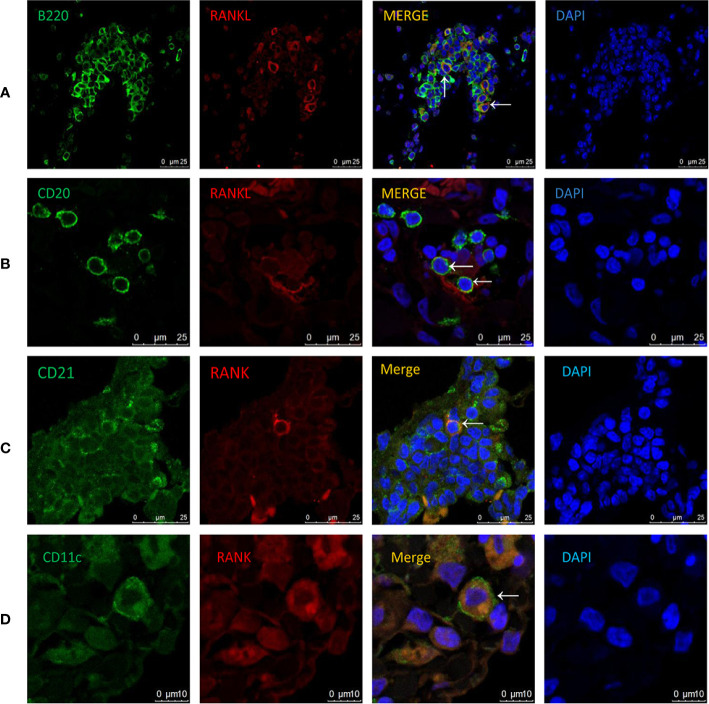
Cellular localization of RANKL and RANK in COPD patients and CS-exposed mice. Coimmunofluorescent staining for RANKL (AlexaFluor 594, red) and B220 (AlexaFluor 488, green) in lungs from CS-exposed mice **(A)**. n = 4; Coimmunofluorescent staining for RANKL (AlexaFluor 594, red) and CD20 (AlexaFluor 488, green) in lungs of COPD patients **(B)**. n = 3; Coimmunofluorescent staining for RANK (AlexaFluor 594, red) and CD21 (AlexaFluor 488, green) in lungs of CS-exposed mice **(C)**. n=4; Coimmunofluorescent staining for RANK (AlexaFluor 594, red) and CD11c (AlexaFluor 488, green) in lungs of COPD patients **(D)**. n = 3; Sections were counterstained with 4′,6-diamidino-2-phenylindole (DAPI) (blue). Arrows indicate double-positive cells. A, B, and C: Scale bar =25 μm. D: Scale bar = 10 μm.

RANKL expression has been detected in various tissues and cells (13–15, 21–23). However, its expression pattern in COPD has not been investigated. To address this, we performed immunofluorescence costaining for RANKL and cellular markers focusing on T and B cells. Interestingly, the majority of RANKL^+^ cells were identified as B cells in CS-exposed mice (**Figure 5A**), while no RANKL expression was detected in T cells (**Supplementary Figure 1**). Human CD20-positive B cells were also found to express RANKL (**Figure 5B**).

To understand the biological effect of B cell RANKL, we further examined the cellular localization of its receptor RANK in the lungs of COPD patients and CS-exposed mice. We found that the RANK^+^ cells were identified as follicular DCs (FDCs) in CS-exposed mice (**Figure 5C**), consistent with previous findings that RANK expression was detectable on the surface of mature DCs (12). We also confirmed the expression of RANK by DCs in human lung tissues (**Figure 5D**).

### Upregulation of RANKL and RANK Expression by IL-17A *In Vitro*

**Figure 6:**
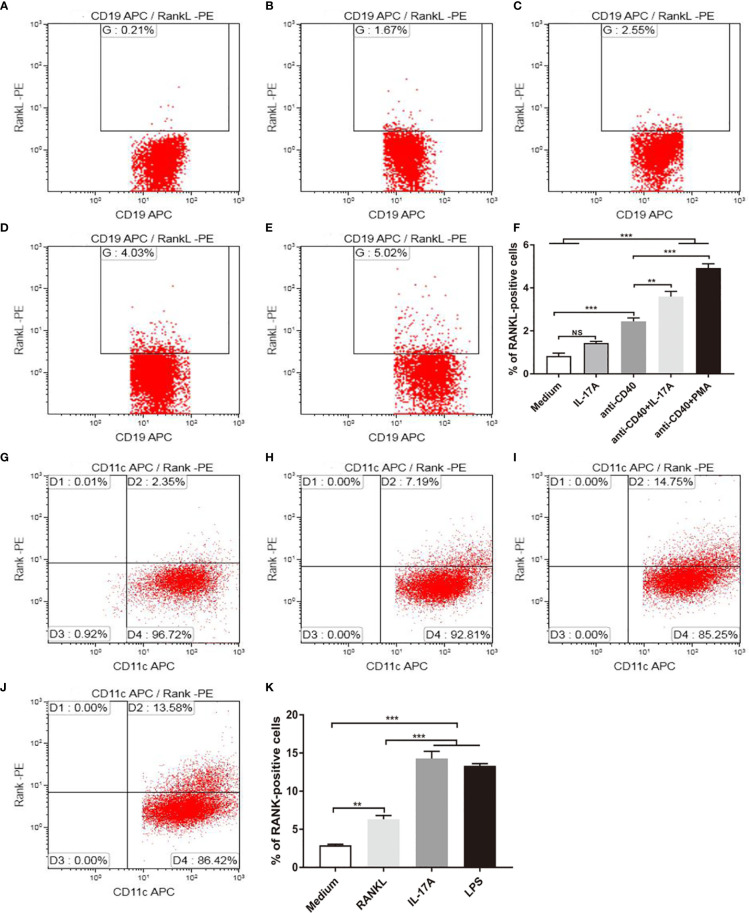
IL-17A promotes RANKL and RANK expression *in vitro*. B cells were stimulated as in **(A)** medium, with **(B)** 100 ng/ml IL-17A, **(C)** 2.5 μg/ml anti-CD40, **(D)** 2.5 μg/ml anti-CD40+100 ng/ml IL-17A, **(E)** 2.5 μg/ml anti-CD40+50 ng/ml PMA, and RANKL expression was detected by flow cytometry. **(F)** The percentage of RANKL positive cells under stimulation; n = 6. BMDCs were stimulated as in **(G)** medium, with **(H)** 100 ng/ml RANKL, **(I)** 100 ng/ml IL-17A, **(J)** 1 μg/ml LPS, and RANK expression was detected by flow cytometry. **(K)** The percentage of RANK positive cells under stimulation; n = 6. Bars indicate mean value, and error bars indicate SEM. **P < 0.01; ***P < 0.001.

Because IL-17A was indispensable for RANKL expression in mice exposed to CS, and RANKL was expressed by B cells, we asked whether IL-17 promoted RANKL expression by B cells *in vitro*. B lymphocytes from spleen of mice were stimulated with recombinant IL-17A, B cell activator anti-CD40 and positive control PMA (**Figure 6E**). B lymphocytes suspended in medium without any stimulus was used as blank control for positive cell gating. The result showed that the percentage of RANKL positive cells measured by flow cytometry was not statistically significantly upregulated in B lymphocytes with IL-17A alone (**Figures 6A, B**), while increased significantly after IL-17A stimulation in activated B lymphocytes *via* CD40 as compared to the CD40-activated B lymphocytes control group (**Figures 6C, D, F**). Since IL-17RA and IL-17RC receptor subunits were both essential for IL-17A signaling (24), we asked whether blocking IL-17A receptors prevented IL-17A–induced RANKL expression by B cells *in vitro*. The result showed that the percentage of RANKL-positive cells measured by flow cytometry was significantly reduced after blocking IL-17RA but not after blocking IL-17RC in CD40-activated B lymphocytes with IL-17A stimulation (**Supplementary Figures 2A–G**).

As DC development takes place in the bone marrow (25), BMDCs were used for *in vitro* study, and were stimulated with recombinant IL-17A, recombinant RANKL and positive control LPS (**Figure 6J**). BMDCs suspended in medium without any stimulus was used as blank control for flow cytometry positive cell gating. As measured by flow cytometry, the proportion of RANK positive cells was increased significantly after IL-17A or RANKL stimulation as compared to the blank control (**Figures 6G–I, K**).

### Upregulation of CXCL13 Gene Expression in DCs Under RANKL Stimulation

**Figure 7:**
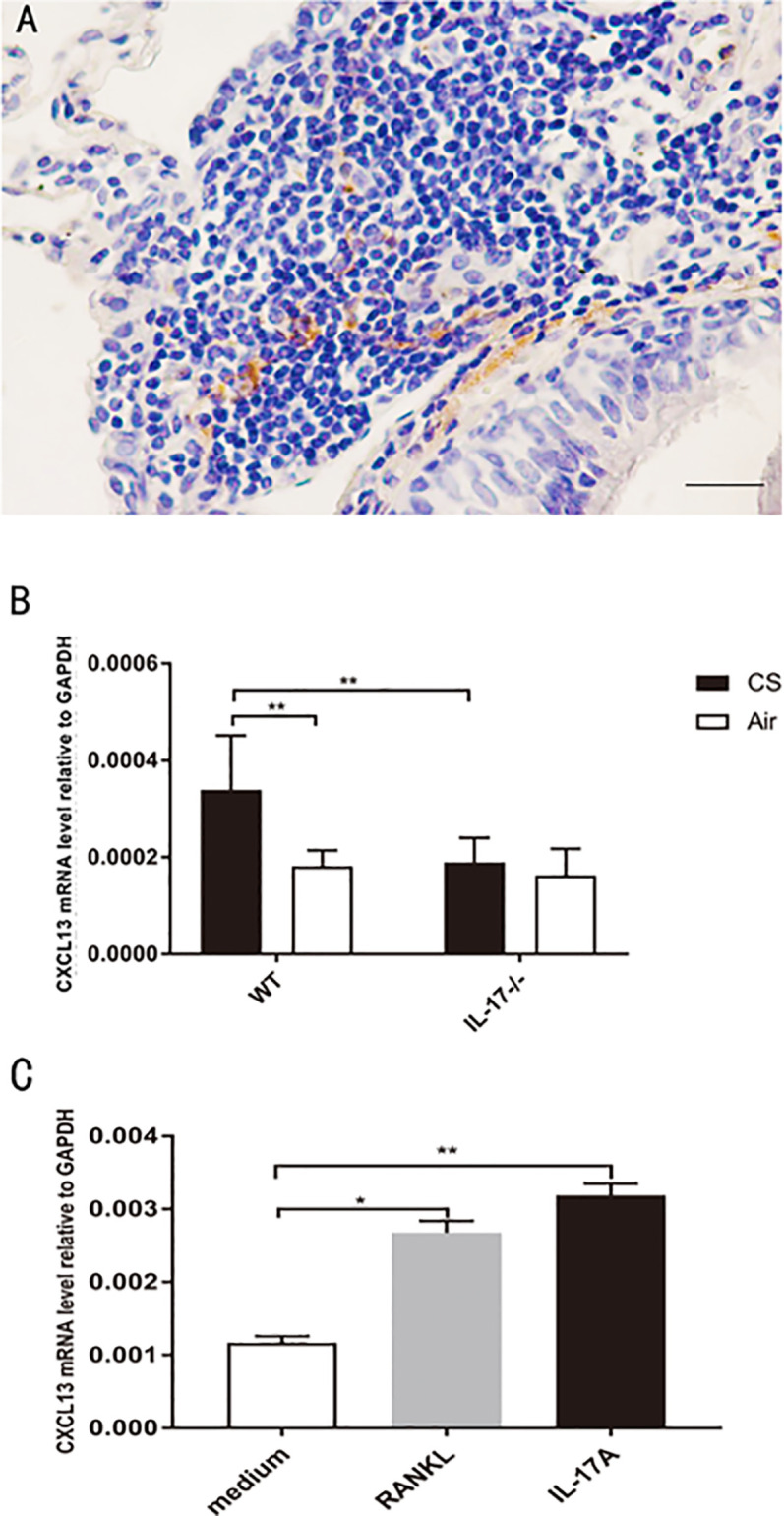
CXCL13 mRNA increases in DCs under RANKL stimulation. **(A)** Immunohistochemical detection of CXCL13 (DAB, brown) in lung lymphoid follicles of a COPD patient. Sections were counterstained with Mayer hematoxylin (blue). Scale bar = 50 μm. **(B)** CXCL13 mRNA in total lung tissue of mice from WT mice and IL17^−/−^ mice; n = 6. **(C)** CXCL13 mRNA in DCs under RANKL and IL-17A stimulation; n = 6. Bars indicate mean value, and error bars indicate SEM. *P <0.05; **P < 0.01.

CXCL13, by recruiting and organizing B lymphocytes, is critical for lymphoid follicle formation in COPD lungs (6, 26, 27). In COPD patients, CXCL13 was expressed within lymphoid follicles (**Figure 7A**). In our animal models, CXCL13 mRNA in total lung tissue was increased in CS-exposed WT mice, but not in air-exposed WT mice and CS-exposed IL17A^−/−^ mice (**Figure 7B**).Our *in vitro* experiments showed that both RANKL and IL-17A increased CXCL13 mRNA in DCs (**Figure 7C**), suggesting that IL-17A, both directly and indirectly *via* RANKL, up-regulated CXCL13 expression.

## Discussion

In this study, for the first time to our knowledge, we demonstrated that lung lymphoid neogenesis in COPD patients was associated with increased expression of RANKL and its receptor RANK, concomitant with upregulation of IL-17A. We further showed that, CS-induced RANKL-RANK expression and the development of lymphoid follicles were dependent on IL-17A in a mouse model of COPD. After identification of RANKL^+^ cells as B lymphocytes and RANK^+^ cells as DCs in lymphoid follicles, we further demonstrated *in vitro* that IL-17A induced RANKL expression in B cells and RANK expression in DCs, while CXCL13, a potent B cell chemoattractant critical for lymphoid follicle formation, was up-regulated in DCs under IL-17A and RANKL stimulation. Thus, our data supported a novel mechanism in CS-induced lymphoid neogenesis in COPD.

Lymphoid follicles are enriched in COPD lung tissues and associate with progression of the disease (4), suggesting a role of adaptive immunity in the pathogenesis of COPD (3, 6). Previous studies have shown that CS-induced lymphoid follicles depend on IL-17, but the cellular and molecular pathways involved are largely unknown, although CXCL12 is implicated (8, 11).

RANKL-RANK pathway has originally been described for their key roles in bone metabolism (14, 15, 28), and later was found to be critical for lymph node homeostasis and lymphoid follicle formation in small intestines (16, 17). We therefore hypothesized that long-term cigarette smoking may induce lung lymphoid neogenesis through upregulation of these molecules *via* IL-17. In the current study, we found increased expression of RANKL and its receptor RANK in lung tissues from COPD smokers as compared to smokers and nonsmokers with normal lung function, and more interestingly, RANKL expression was prominent within lymphoid follicles. This novel finding was recapitulated in a well-established mouse model, which revealed the involvement of IL-17–dependent RANKL expression in lymphoid neogenesis induced by CS exposure.

RANKL expression has been detected in various tissues and cells, including T cells (13), B cells (21), osteoblasts (OBs), osteocytes and bone stroma, and the lung (14, 15, 22, 23). In this study the majority of RANKL^+^ cells were identified as B cells within lymphoid follicles in CS-exposed mice, and RANKL was also detected in B cells in human lung tissues. Interestingly, RANK expression was also remarkably increased in lymphoid follicles in lung tissues from COPD patients and the mouse model. We further localized the expression of RANK on DCs, which was consistent with a previous report (12). GeurtsvanKessel and colleagues found earlier that DCs were crucial for maintenance of tertiary lymphoid structures in the lung of influenza virus-infected mice *via* secreted lymphotoxin (LT) beta and homeostatic chemokines (CXCL-12, CXCL-13, CCL-19, and CCL-21) (29). Taken together these findings pointed to a mechanistic link between B cells and DCs *via* RANKL pathway in the development and/or maintenance of tertiary lymphoid structures in COPD.

It is not surprising to see that RANKL expression is dependent on IL-17 in our mouse model, as there has been evidence to show that IL-17 mediates RANKL expression in fibroblasts or osteoblasts (18), mouse neutrophils (30), and human periodontal ligament cells (31). In addition, IL-17 was also capable of promoting B cell activation (32). Our *in vitro* study confirmed that IL-17A stimulated RANKL expression in B cells and blocking IL-17RA, one of the IL-17A receptor subunits, downregulated IL-17A–induced RANKL expression. Both IL-17 and RANKL also increased the expression of RANK in DCs, confirming the *in vivo* findings.

The role of DCs in the development of lymphoid follicles induced by cigarette smoking is yet to be determined. CXCL13 is a key molecule in lymphoid follicle formation in COPD lungs (6). It binds to CXCR5 expressed on B cells and follicular T helper (Tfh) cells, and then recruit and organize B and T lymphocytes (6, 25, 26). It is well established that secretion of CXCL13 by FDCs plays an important role in the recruitment of both CXCR5^+^ B cells and CXCR5^+^ CD4 T cells into the FDC network region (33). Sputum CXCL13 protein levels and CXCL13 mRNA transcripts in whole lung were increased in COPD patients compared with samples from never-smoker controls, and neutralization of CXCL13 in CS-exposed mice reduced the number of organized lymphoid follicles (6). IL-17 promoted the expression of CXCL13 in LPS-induced lymphoid follicles in mice (10). In the current study, we found increased CXCL13 mRNA transcripts in the whole lung of CS-exposed WT mice, but not in CS-exposed IL-17A^−/−^ mice and air-exposed WT mice. IL-17A was shown to promote CXCL13 expression in various cells (34, 35). Interestingly, RANKL was also shown to be able to promote FDCs to express higher levels of CXCL13 (17). In our *in vitro* experiments, IL-17A or RANKL stimulation upregulated CXCL13 mRNA in DCs. Thus it can be proposed that B cells, by production of RANKL in the milieu of IL-17, stimulates DCs to express CXCL13 which in turn recruits and organizes B lymphocytes, creating a self-perpetuating loop in lymphoid neogenesis induced by CS.

A limitation of our study is the lack of direct evidence of the function of RANKL. Previous studies found that RANKL-deficient mice showed severe osteopetrosis, with no osteoclasts, marrow spaces, or tooth eruption, and exhibited profound growth retardation at several skeletal sites, including the limbs, skull, and vertebrae (36, 37) , and therefore it seems unsuitable to use RANKL-deficient mice for long-term CS exposure. Further studies using antibodies against RANKL may be needed to elucidate its role in this model.

**Figure 8:**
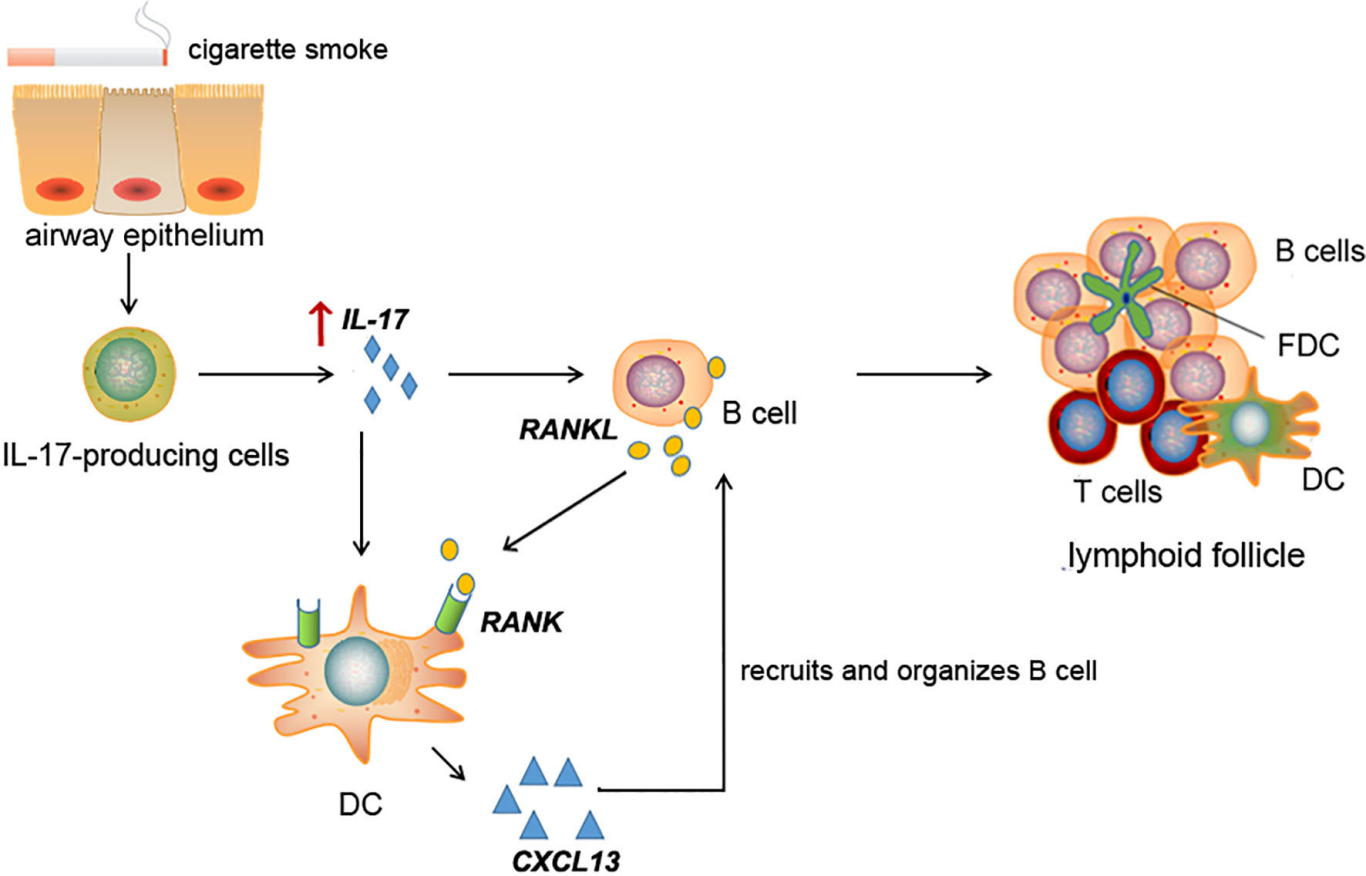
Schematic diagram for cigarette smoke-induced lymphoid neogenesis in COPD involves IL-17/RANKL pathway. IL-17 is elevated in lung tissues of COPD patients or cigarette smoke-exposed mice. Elevated IL-17 promotes RANKL expression in B cells and RANK expression in DCs. CXCL13, a potent B cell chemoattractant, was up-regulated in DCs under RANKL and IL-17 stimulation, which in turn contribute to lymphoid follicle formation.

In conclusion, the present study found increased RANKL and its receptor RANK expression, along with IL-17A, in lung tissues of COPD patients. Long-term CS exposure-induced lymphoid follicle formation and RANKL/RANK expression were dependent on IL-17A. In the lymphoid follicles, RANKL^+^ cells were mostly B cells, while RANK was expressed by DCs, both of which were responsive to IL-17A stimulation. Lymphoid follicle B cells, *via* the RANKL pathway, may create a positive feedback loop by upregulating CXCL13 expression in DCs (**Figure 8**). Taken together, our study reveals a novel mechanism of IL-17/RANKL pathway in lymphoid neogenesis induced by CS exposure, shedding new light on the understanding of adaptive immunity in pathogenesis of COPD.

## Data Availability Statement

The raw data supporting the conclusions of this article will be made available by the authors, without undue reservation.

## Ethics Statement

The studies involving human participants were reviewed and approved by Ethics Committee of Peking University Third Hospital and Beijing Tongren Hospital, Capital Medical University. The patients/participants provided their written informed consent to participate in this study. The animal study was reviewed and approved by Ethics Committee of Peking University Third Hospital and Beijing Tongren Hospital, Capital Medical University.

## Author Contributions

Conception and design: JX, LZ, JT, XY, and YS. Acquisition of data: LZ, JX, JT, XY, YLi, and YS. Help with animal experiment: JX, YLe, and YR. Analysis and interpretation: JX, LZ, JT, XY, RJ, and YS. Drafting the manuscript for important intellectual content: JX, LZ, and YS. All authors contributed to the article and approved the submitted version.

## Funding

This work was supported by the National Natural Science Foundation of China (81470239, 81770040, 81970041).

## Conflict of Interest

The authors declare that the research was conducted in the absence of any commercial or financial relationships that could be construed as a potential conflict of interest.

## Abbreviations

IL-17, interleukin 17; RANKL, receptor activator of nuclear factor-κB ligand; RANK, receptor activator of nuclear factor-κB; DC, dendritic cell; FDC, follicular dendritic cell.
