# Supplementary material for: Cigarette Smoke-Induced Lymphoid Neogenesis in COPD Involves IL-17/RANKL Pathway
Source: Front Immunol. 2021 Feb 5;11:588522. doi: 10.3389/fimmu.2020.588522 (PMC7892459; doi:10.3389/fimmu.2020.588522)
Supplement: Supplementary file 1 [file DataSheet_1.docx]

**Cigarette smoke-induced lymphoid neogenesis in COPD involves IL-17/RANKL pathway**

**ONLINE DATA SUPPLEMENT**


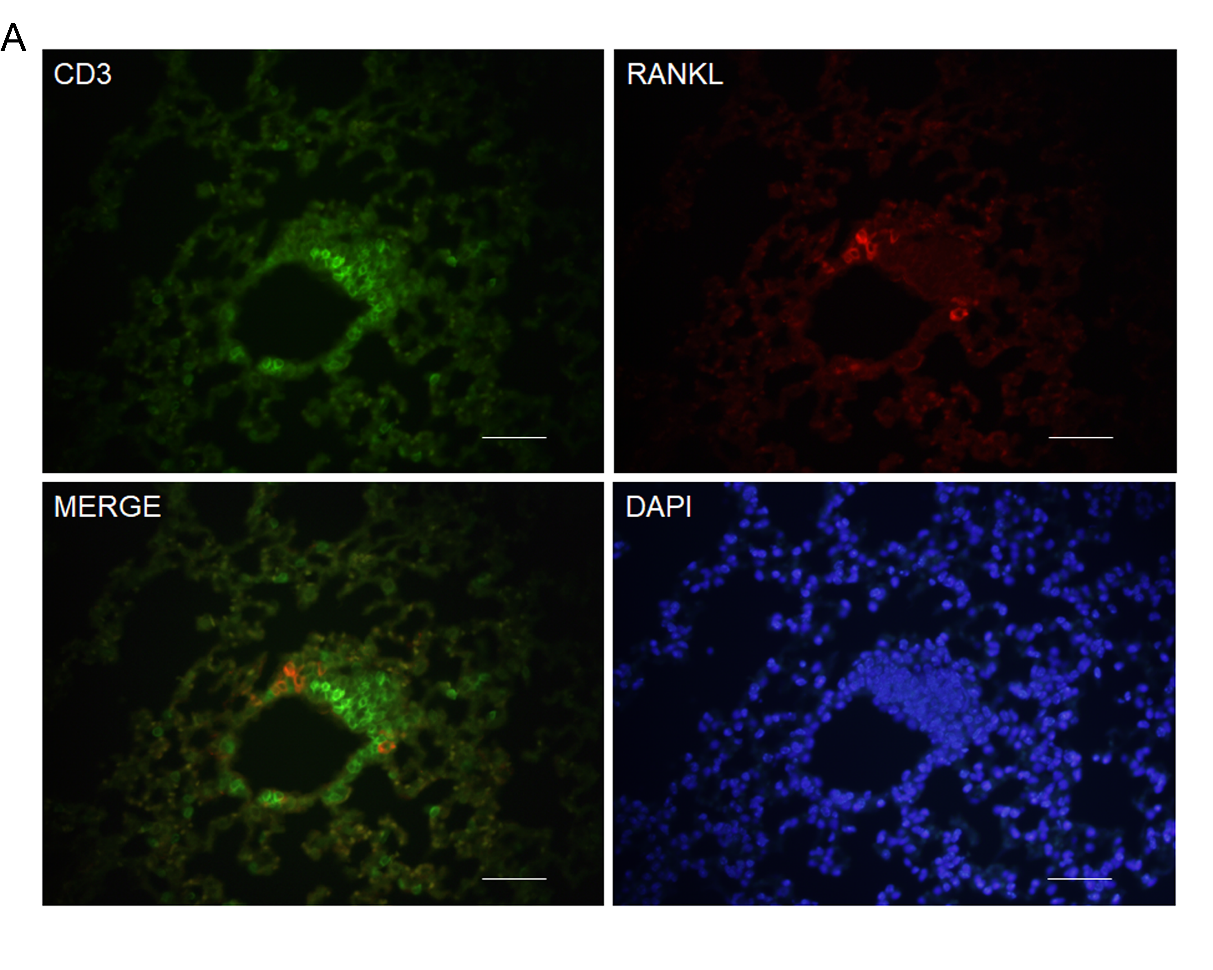
**Supplementary Figure 1**

**Supplementary Figure 1:** **Cellular localization of RANKL is not detected on T cells in CS-induced lymphoid neogenesis.** (A) Coimmunofluorescent staining for RANKL (AlexaFluor 594, red) and CD3 (AlexaFluor 488, green) in lungs from CS-exposed mice. Scale bar =50 μm

**Supplementary Figure 2**


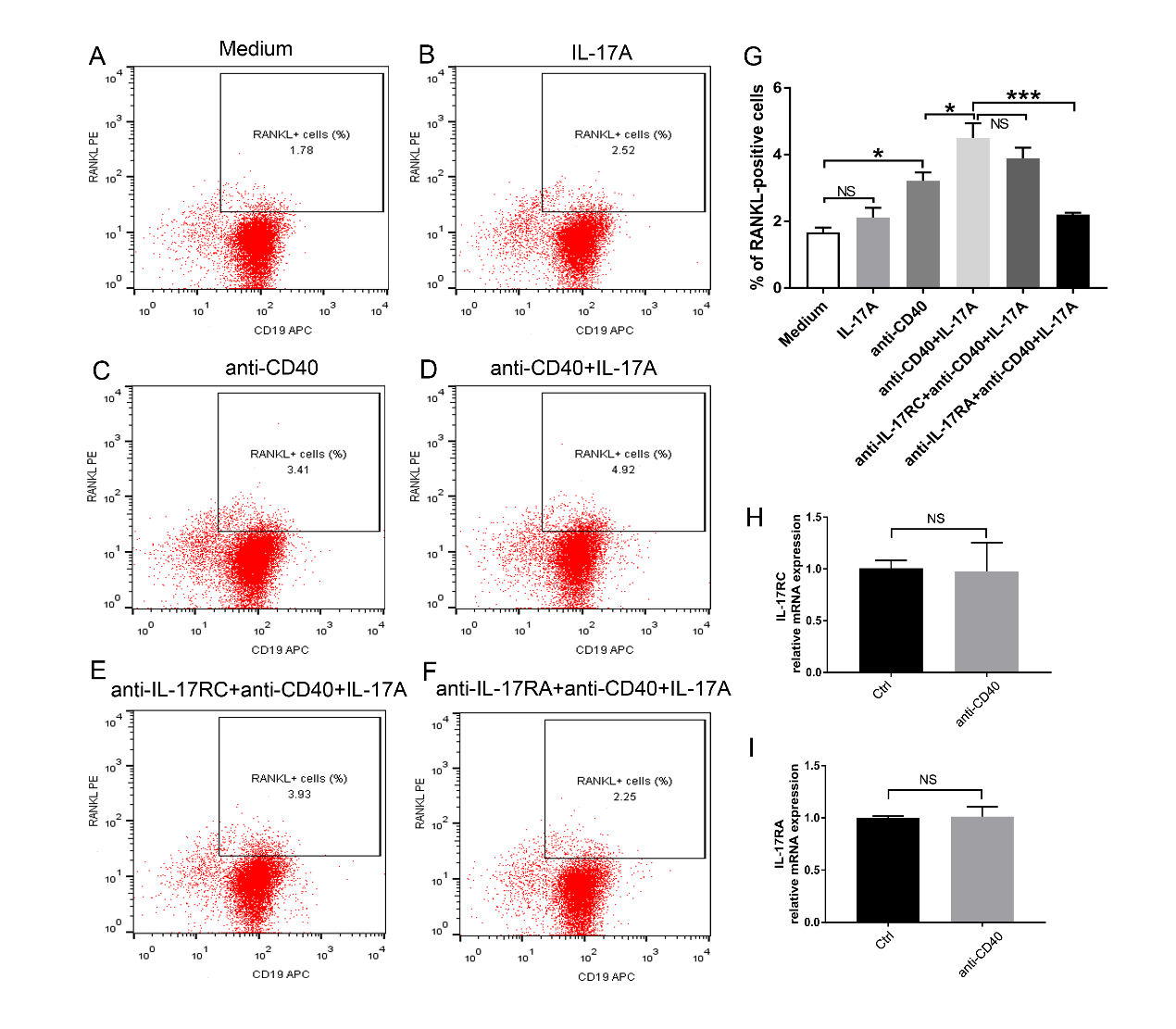


**Supplementary Figure 2: IL-17RA is involved in IL-17A-induced RANKL expression in B cells.** B cells were stimulated for 48h as in (A)medium, with (B) 100ng/ml IL-17A, (C) 2.5μg/ml anti-CD40, (D) 2.5μg/ml anti-CD40+100ng/ml IL-17A, (E) 2.5μg/ml anti-IL-17RC+ 2.5μg/ml anti-CD40+100ng/ml IL-17A, (F) 1.5μg/ml anti-IL-17RA+ 2.5μg/ml anti-CD40+100ng/ml IL-17A, (G)The percentage of RANKL positive cells under stimulation; n = 4. (H-I) IL-17RC mRNA and IL-17RA mRNA in B cells with 2.5μg/ml anti-CD40 ; n=3. Bars indicate mean value, and error bars indicate SEM. NS indicate [no](javascript:;) [statistical](javascript:;) [differences](javascript:;),*P <0.05; ***P<0.001.
